# Gene Fusion Analysis in the Battle against the African Endemic Sleeping Sickness

**DOI:** 10.1371/journal.pone.0068854

**Published:** 2013-07-17

**Authors:** Philip Trimpalis, Vassiliki Lila Koumandou, Evangelia Pliakou, Nicholas P. Anagnou, Sophia Kossida

**Affiliations:** Bioinformatics & Medical Informatics Team, Biomedical Research Foundation, Academy of Athens, Athens, Greece; Hospital for Sick Children, Canada

## Abstract

The protozoan *Trypanosoma brucei* causes African Trypanosomiasis or sleeping sickness in humans, which can be lethal if untreated. Most available pharmacological treatments for the disease have severe side-effects. The purpose of this analysis was to detect novel protein-protein interactions (PPIs), vital for the parasite, which could lead to the development of drugs against this disease to block the specific interactions. In this work, the Domain Fusion Analysis (Rosetta Stone method) was used to identify novel PPIs, by comparing *T. brucei* to 19 organisms covering all major lineages of the tree of life. Overall, 49 possible protein-protein interactions were detected, and classified based on (a) statistical significance (BLAST e-value, domain length etc.), (b) their involvement in crucial metabolic pathways, and (c) their evolutionary history, particularly focusing on whether a protein pair is split in *T. brucei* and fused in the human host. We also evaluated fusion events including hypothetical proteins, and suggest a possible molecular function or involvement in a certain biological process. This work has produced valuable results which could be further studied through structural biology or other experimental approaches so as to validate the protein-protein interactions proposed here. The evolutionary analysis of the proteins involved showed that, gene fusion or gene fission events can happen in all organisms, while some protein domains are more prone to fusion and fission events and present complex evolutionary patterns.

## Introduction

African trypanosomiasis is one of the neglected parasitic diseases which infects both humans and animals in regions of sub-Saharan Africa which cover about 37 countries; more than 60 million people are at risk even today [1]. The disease is caused by protozoa of the species *Trypanosoma brucei* and is transmitted by the tsetse fly, through a bite into the bloodstream. The infection spreads throughout the body and, if untreated, can be lethal. The symptoms are sometimes ignored or at least underestimated, as they include fever and other common symptoms, and behavioral changes, such as anxiety or sleep disorders. Unfortunately, the tests used to verify the infection nowadays include painful and complicated procedures such as lumbar puncture, and lymph node aspiration. The available drugs, as effective as they may be, are outdated and can cause severe and often deadly side-effects [2].

Sustained infection is caused by the unique ability of the Trypanosomes to deceive the host’s immune system through the antigenic variation of its surface proteins [3], making it nearly impossible for vaccination to succeed. Some efforts, however, are focusing on the identification of compounds that are presented on the parasite’s surface and remain unchanged, which can serve as therapeutic targets, using the promising DNA vaccination technology developed recently [4].

Another approach to this problem is the possible identification of protein-protein interactions (PPIs) which, if inhibited pharmacologically, can cause lethality to the parasite through the blockage of a necessary biological pathway which cannot be bypassed in any other way. A preliminary study [5] identified some potential such interactions, using domain fusion analysis to compare the *T. brucei* proteome to the proteomes of a number of other protists. Here we have extended this analysis to include another 19 fully sequenced organisms, covering the full range of evolutionary diversity in eukaryotes and prokaryotes. For all proteins involved in the PPIs identified by this method, we examine their involvement in crucial metabolic pathways and their evolutionary history.

## Methods

In order to detect PPIs, we used the domain fusion analysis (otherwise known as gene fusion analysis, or Rosetta stone method) to identify potential protein pairs that possibly interact, and evaluate these targets considering their importance for survival of the pathogen.

**Figure 1:**
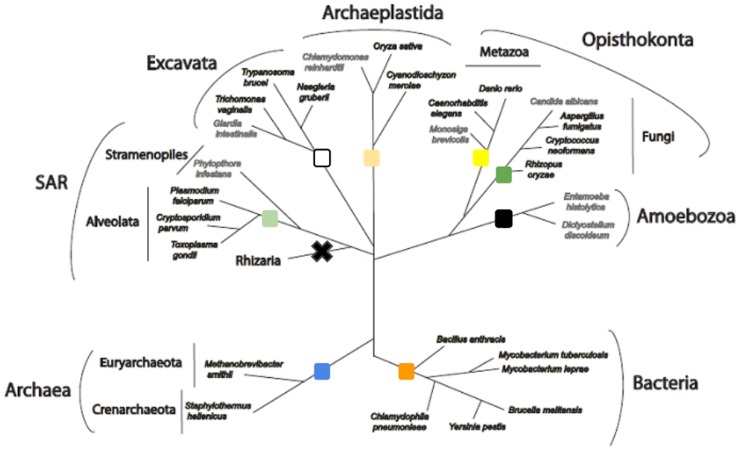
Evolutionary relationships of the organisms used in this study. The selected organisms represent all major eukaryotic and prokaryotic lineages along the tree of life. Colored boxes in the different tree nodes correspond to the colors used in Table 1. Species names shown in grey were examined in a previous study [5] based on which we have excluded the Rhizaria from the analysis as there is not enough sequence data and the Amoebozoa as the two completed available genomes have already been analyzed for fusion events by this method. The tree is based on the model proposed by Dacks and Field [49]; the tree is schematic i.e. the order of the branching events delineating organismal relationships is retained, but distances are not drawn to scale for clarity of presentation.

The domain fusion analysis has already been successfully applied to the specific pathogen [5] to identify protein-protein interactions that can be specifically inhibited, making the pathogen unable to reproduce, or to survive, within the host organism. Recently, a software tool to make this process automated has been published [6] so the analysis can now be performed with more organisms. Overall, 19 organisms, both pathogenic and non-pathogenic for humans, were used in this study (Table 1, Figure 1) to compare with *T. brucei* in order to have more results that can be approached pharmacologically.

**Table 1:** Details of the organisms used in this study.

Organism	Strain	Proteome size	Number of genes	Source	Organism taxonomy	Characterization	Unique events	Verified events
*Trypanosoma brucei*	TREU927	8,788	9,068 [29]	NCBI	Excavates	sleeping sickness (p)	–	–
*Trichomonas vaginalis*	G3	59,681	∼60,000 [30]	NCBI	Excavates	trichomoniasis (p)	13	0
*Mycobacterium tuberculosis*	CDC1551	4,189	4,294 [31]	NCBI	Bacteria	tuberculosis (p)	4	0
*Mycobacterium leprae*	TN	1,605	2,720 [32]	NCBI	Bacteria	Hansen’s disease (p)	4	1
*Yersinia pestis*	KIM10	4,205	4,457 [33]	NCBI	Bacteria	plague (p)	6	1
*Bacillus anthracis*	Sterne	5,287	5,287 [34]	NCBI	Bacteria	anthrax (p)	9	2
*Brucella melitensis*	16M	3,199	3,198 [35]	NCBI	Bacteria	ovine brucellosis (p)	4	1
*Chlamydophila pneumoniae*	AR39	1,116	1,052 [36]	NCBI	Bacteria	pneumonia (p)	1	1
*Methanobrevibacter smithii*	ATCC 35061	1,795	1,795 [37]	NCBI	Archaea	human gut archaeon	3	1
*Staphylothermus hellenicus*	DSM 12710	1,599	1,599 [38]	NCBI	Archaea	thermophile	3	1
*Plasmodium falciparum*	3D7	5,336	∼5,300 [39]	NCBI	Chromalveolates	malaria (p)	8	1
*Cryptosporidium parvum*	Iowa	3,805	3,807 [40]	NCBI	Chromalveolates	cryptosporidiasis (p)	3	0
*Toxoplasma gondii*	ME49	7,993	8,032 [41]	ToxoDB	Chromalveolates	toxoplasmosis (p)	13	2
*Oryza sativa*	Nipponbare	35,584	37,544 [42]	NCBI	Archaeplastida	asian rice	18	3
*Cyanidioschyzon merolae*	10D	5,016	5,331 [43]	biol.s.u-tokyo.ac.jp	Archaeplastida	ancient red algae	8	4
*Caenorhabditis elegans*	nematode	25,433	>19,000 [44]	NCBI	Metazoa	nematode	26	8
*Danio rerio*	AB	29,499	35,156 [45]	UniProt	Metazoa	zebra fish	23	9
*Cryptococcus neoformans*	JEC21	6,594	∼6,500 [46]	NCBI	Fungi	cryptococcosis (p)	8	2
*Rhizopus oryzae*	RA 99–880	17,459	17,467 [47]	Broad Institute	Fungi	organic matter fungus	14	8
*Aspergillus fumigatus*	Af293	9,888	9,926 [48]	Broad Institute	Fungi	organic matter fungus	12	4
							180	49

For each species, the database used as the source of the data is shown, the strain that the data corresponds to, as well as the number of genes estimated for each genome, and the number of protein sequences annotated for each proteome at the specific database source. Pathogenic organisms are indicated by (p) and the disease they cause is shown. Finally, the number of fusion events detected by the SAFE software and verified in this study is given.

Following the identification of potential interacting protein pairs, we used phylogenetic trees to determine the evolutionary fate of each protein pair associated with a putative PPI, in order to focus on protein pair candidates that are fused in the host organism (*Homo sapiens*). Theoretically, inhibiting PPIs that are unique to the parasite and not shared by the host, allows us to make a significant step towards the absence of severe side effects, if a future drug is produced to block the specific interaction.

To perform the analysis, we used a workflow that included:

- The automatic identification of fusion events which can then be assigned to the respective PPIs, through the SAFE platform [6] with the following parameters: removal of duplicate proteins from the proteome: 85%, minimum length of a functional domain: 70AA (100AA is the average length of a protein domain [7]), minimum BLAST % identity (same AA) per domain: 27% (below that level, homology cannot be safely concluded [8]), minimum fused protein coverage: 70%, maximum domain overlap: 0AA (no overlap allowed), multiple protein results: 5 proteins, e-value cutoff: 9*10^−3^. These parameters were set to these numbers as they yield better quality results as observed from previous analyses of this kind, and were therefore implemented here as well.
- The backward BLAST process, used as a confirmation step for the fusion events [9]. According to this process, the two separate proteins found to participate in a fusion event, must correspond to the fused protein as the best reciprocal BLAST hit.
- To study the evolutionary history of the protein pairs, the identified fused protein was used as a BLAST query to search for homologs against the major organism lineages in order to observe the evolutionary pattern of each fusion event (fusion, fission, etc.). Within each organism family group, we not only checked the BLAST hit with the highest identity value and the lowest E-value threshold (as described previously [5]), but collected data about all the top hits. These results were then mapped onto a schematic phylogenetic tree, showing the relationships between these organism groups (Figure 1). The state of each protein in *Naegleria gruberi,* a relatively close neighbor to *T. brucei*, was also checked in order to better refine the evolutionary history of each event within the excavates, and to distinguish kinetoplastid-specific events. In certain organisms, both fused and separate configurations of the proteins were found with equivalent scores, and these are marked with ‘f/s’. This analysis also allowed us to focus on the results that appear fused in the host organism (*Homo sapiens*). If a protein pair is separated in *T. brucei* but fused in the host, and if the predicted protein-protein interaction is crucial to the parasite’s survival, designing an inhibitor for the protein-protein interaction would specifically target the parasite and not the host protein; this marks the identified interaction as a promising drug candidate.
- For the last, but most crucial step, the proteins involved in the fusion events that presented the respective PPIs, were checked bibliographically for already reported interactions. For the results that were not previously reported as interacting protein pairs, including proteins that were hypothetical, we searched in the Conserved Domain Database (CDD) [10] to identify conserved functional domains. For the molecular characterization of all protein pairs, we also searched the KEGG (Kyoto Encyclopedia of Genes and Genomes) database to identify the metabolic pathway in which the suggested interaction takes place. Also, Gene Ontology annotation as found in UniProt was used for the classification of the protein pairs identified by the gene fusion analysis.

## Results and Discussion

**Figure 2:**
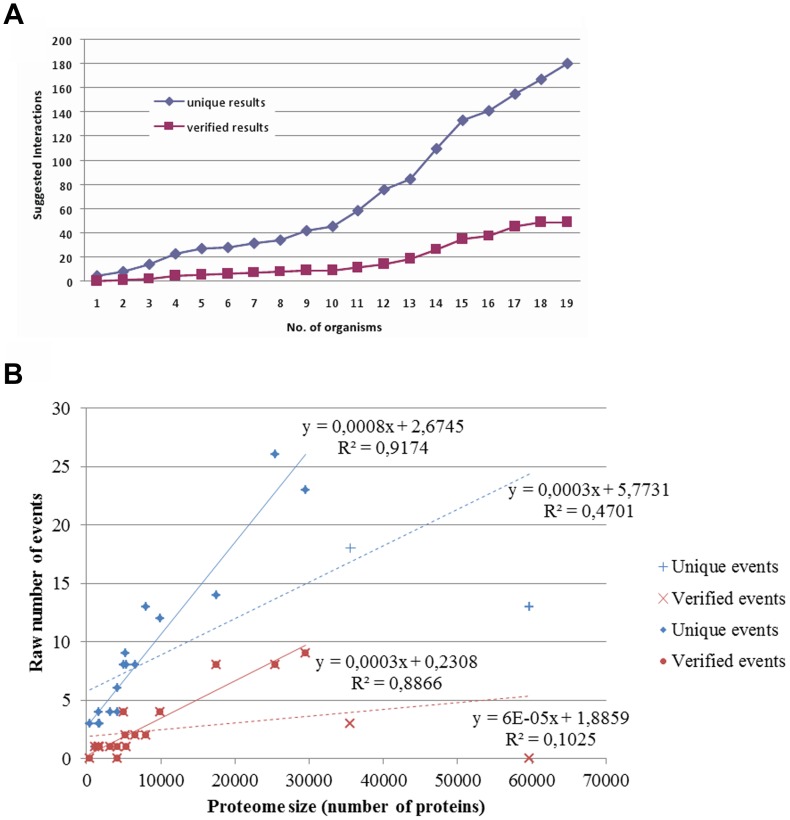
The number of detected fusion events in *T. brucei* increases as more organisms are used, and with increasing genome size. Panel A: The cumulative number of protein-protein interactions suggested by the detected fusion events in this study is shown, as the number of organisms used to detect such events increases. Panel B: The number of fusion events detected per organism in this study is shown, relative to the proteome size of each organism. There is a rough linear correlation, which improves markedly if the data for the largest two proteomes of *Trichomonas vaginalis* (60,000 proteins) and *Oryza sativa* (∼35,000 proteins) are excluded as outliers. Note that the low number of events found in these two proteomes may be due to the high number of transposable elements and repetitive sequences that they contain (up to 65%) [30], [50]. The dashed blue line corresponds to all unique events, whereas the solid blue line corresponds to the unique events excluding the largest two proteomes; the dashed red line corresponds to all verified events, whereas the solid red line corresponds to the verified events excluding the largest two proteomes.

In total, 180 fusion events were automatically detected by the SAFE software, from which, only 49 passed the backward BLAST verification step and were thereafter considered for a proposed PPI (Table S1). Overall, we observed that the more proteomes we used for the comparison with *T. brucei*, the more fusion events were identified (Figure 2A). Additionally, the number of events found in each organism shows a positive correlation, in general, with the size of the proteome examined (Figure 2B), as has been described previously [9], [11], [12].

The 49 verified events, which passed the backward BLAST verification step (Table S1), represent 39 unique protein pairs, as some of them are found multiple times by the SAFE software when analyzing different organisms (for example, the fusion of the DNA topoisomerase IB small and large subunit was detected by SAFE both in *C. merolae* and in *D. rerio*; identical fusions in different organisms are highlighted in Tables S2, S3 and S4).

**Figure 3:**
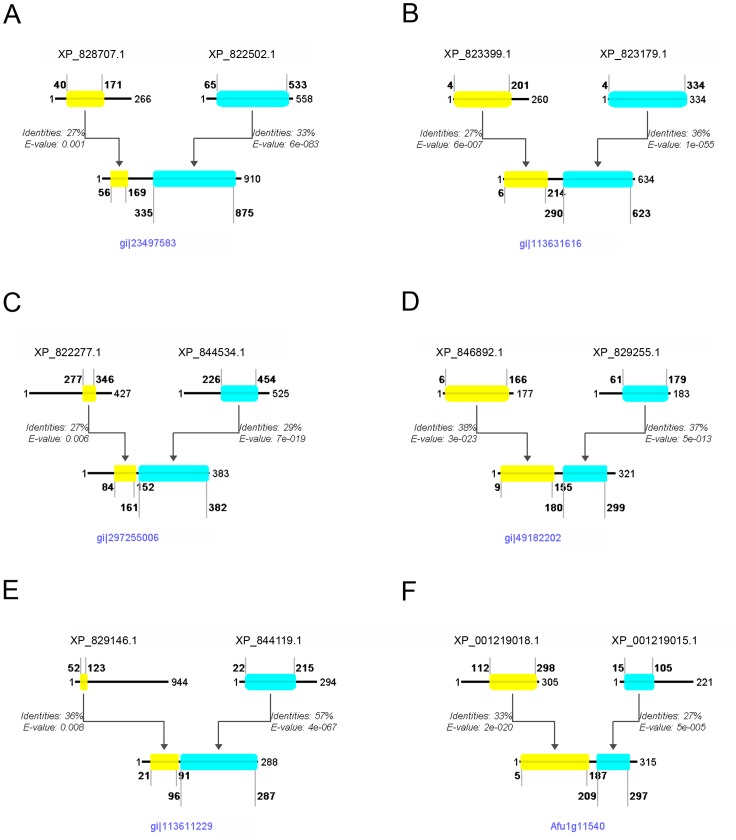
Selected gene fusions identified in this study. Schematic alignment of the *T. brucei* protein pair with the fused protein in another organism, showing the amino acid positions that delineate the beginning and end of the alignment, relative to the full protein length, as well as the % identity and the E-value given by BLAST for each alignment. Panel A: G6PD-6PGL Bifuctional enzyme fusion, detected in *P. falciparum*. Panel B: NAD oxidoreductase fusion detected in *O. sativa*. Panel C: Centromere binding protein -nucleolar protein fusion, detected in *S. hellenicus*. Panel D: Peptide Methionine Sulfoxide Reductase (PMSR) fusion, detected in *B. anthracis*. Panel E: Protein kinase ck2 regulatory subunit - hypothetical protein fusion, detected in *O. sativa*. Panel F: CHORD-SGT1 domains fusion, detected in *A. fumigatus*. Further details are discussed in the text.

These protein pairs were further categorized based on their functional domain annotations. Six protein pairs correspond to hypothetical proteins, for which only limited domain or similarity information is available (Table S3). 15 fusion events representing 12 unique protein pairs are composed of one functionally annotated protein and one hypothetical (Table S4). 28 fusion events representing 24 unique protein pairs (4 of which are all DNAJ chaperone protein pairs) are composed of two functionally annotated proteins (Table S2). 21 of the identified protein pairs (17 distinct protein pairs) participate in the same pathway, based on their functional annotation (marked with [p] in the description column in Table S2). 10 of the identified protein pairs (6 distinct protein pairs) have already been reported as PPIs in the literature, or they form part of the same protein complex (highlighted yellow in the description column in Table S2). These results demonstrate the credibility of the fusion analysis method but are not discussed further. We discuss selected results below (Figure 3), representing both annotated and hypothetical proteins (excluding experimentally verified PPIs, and protein pairs that belong to the same protein family or same pathway).

The 49 results that passed the backward BLAST verification, were also checked for Gene Ontology (GO) annotation. As some of these were found multiple times, the common events were reduced to one before the GO analysis. For these 39 unique protein pairs, the Gene Ontology search did not show any significant bias for the biological process of the proteins involved in the fusion events in general, nor for their respective molecular function (Table S5). Also, these events were classified according to the cellular component attributed from the annotation. Based on the GO annotations, approximately 40% of our results had unknown biological process (compared to ∼71% for the whole genome), 9% of our results had unknown molecular function (compared to ∼39% for the whole genome), and 59% of our results had unknown cellular component (compared to ∼47% for the whole genome).

### Examples of Fusions Involving Functionally Annotated Proteins

#### G6PD-6PGL Bifunctional enzyme fusion

*Detected in Plasmodium falciparum (GI: 23497583).* The bifunctional nature of the G6PD-6PGL enzyme in *Plasmodium falciparum* was elucidated some years ago and is known for its unique structural and functional characteristics which are restricted to this genus [13], [14]. These silencing experiments have shown the important role of the enzyme in the infection cycle of the parasite, and together with the gene being only partly homologous to humans, it was proposed as an ideal target for therapeutic strategies [13]. This enzyme in *T. brucei* is a complex of two separate proteins (XP_828707.1 and XP_822502.1, Figure 3A), that work together to achieve the same result, whereas the pair is fused in humans (Table S2). A potential inhibitor for such an interaction could thus be a potential drug candidate for trypanosomiasis.

#### NAD oxidoreductase fusion

*Detected in Oryza sativa (GI: 113631616)*. In this fusion we detected an NAD(P) oxidoreductase (accession number: XP_823399.1) fused with another oxidoreductase (accession number: XP_823179.1) (Figure 3B). The first oxidoreductase contains a Rossmann-fold NAD(P) binding domain, which is often found in MDR proteins (Medium chain Reductase/Dehydrogenases) as a C-terminal domain, paired with an N-terminal catalytic domain homologous to the second oxidoreductase involved in this fusion event. This shows a unique structure in *Oryza sativa* with the fusion of the two proteins, which is not observed in *T. brucei* and other higher eukaryotes, such as *Homo sapiens* (Table S6).

#### Centromere binding protein - nucleolar protein fusion

*Detected in Staphylothermus hellenicus (GI: 297255006).* In this fusion we detected a centromere binding protein (accession number: XP_822277.1) fused with a nucleolar protein (accession number: XP_844534.1) (Figure 3C). The centromere binding protein contains a PUA domain which is predicted to bind RNA. The PUA domain, named after Pseudouridine synthase and Archaeosine transglycosylase, was detected, among others, in archaeal and eukaryotic pseudouridine synthases belonging to a family of predicted ATPases that may be involved in RNA modification. The nucleolar protein contains a typical S-adenosyl methyl-transferase (SAM) domain. This domain is known to supply, in some cases [15], methyl groups to uridine tRNAs, allowing the pseudouridine synthases to proceed with the RNA modification from uridine to pseudouridine [16]. There could be a simple explanation for these two domains found fused in a single protein with a Sun (Sad1 - UNC) domain architecture. The fused protein might have increased efficiency by making use of the PUA RNA-binding motif to bind RNA molecules specifically and carry out the pseudouridine synthase modification.

### Examples of Fusions Involving Functionally Annotated Proteins and Hypothetical Proteins

#### Peptide Methionine Sulfoxide Reductase (PMSR) fusion

*Detected in Bacillus anthracis (GI: 49182202) and Methanobrevibacter smithii (GI: 148551659).* In this fusion we detected a peptide methionine sulfoxide reductase (PMSR) protein (accession number: XP_846892.1) fused with a hypothetical protein (accession number: XP_829255.1) (Figure 3D). The PMSR protein contains a homonymous domain, the function of which is to reduce the critical oxidized methionine sulfoxide residues in proteins to methionine. However, recent studies show that mammals use methionine-S-sulfoxide reductase (MsrA) to reduce methionine-S-sulfoxide, and are unable to reduce the methionine-R-sulfoxide isoform [17]. Notably, the second protein involved in this fusion event, contains the SelR domain that is used to perform the reduction of the R isoforms of methionine sulfoxide [18]. Thus, this allows us to suppose that these two domains, working together, can achieve the reduction of both stereoisomers of methionine sulfoxide. Additionally, the fact that these two are found fused together, uncovers a genetic tension to incorporate the function of these two separate proteins into one single protein.

#### Protein kinase ck2 regulatory subunit – hypothetical protein fusion

*Detected in O. sativa (GI: 113611229).* In this fusion we detected a Casein kinase II regulatory subunit (accession number: XP_829146.1) fused with a hypothetical protein (accession number: XP_844119.1) (Figure 3E). The Casein kinase, is a ubiquitous, well-conserved protein kinase involved in cell metabolism and differentiation. It is characterized by its preference for Ser or Thr in acidic stretches of amino acids. The beta-subunit is believed to be regulatory, possessing an N-terminal auto-phosphorylation site, an internal acidic domain, and a potential metal-binding motif [19]. The hypothetical protein on the other hand, contains a domain of unknown function (DUF3451) as well as a C2 domain, which is a Ca^2+^-dependent membrane-targeting module also found in protein kinase C; C2 domains are often found coupled to enzymatic domains, e.g. of the PTEN phosphatase and the PI3-kinase [20]. We propose that the resulting fusion protein aids the correct localization of the casein kinase, and thus its signalling activity. This protein pair is found fused in human and separate in *T. brucei* (Table S4), so it has the potential to be a good drug target.

#### CHORD – Sgt1 domains fusion

Detected in D. rerio (Uniprot Accession No: Q6DBR7), R. oryzae (Gene number: RO3T_16834), A. fumigatus (Gene number: Afua_1g11540). In this fusion we detected a phosphatase-like protein (accession number: XP_001219018.1) fused with a hypothetical protein (accession number: XP_001219015.1) (Figure 3F). The hypothetical protein contains two CHORD domains (Cysteine and Histidine-Rich Domain), which are common to a family of highly conserved proteins known as CHPs (CHORD-containing proteins). These proteins were recently reported to play important roles in plant disease resistance, and homologous protein complexes in animals are involved in fighting microbial infections [21]. In plants, the CHPs interact with Sgt1 and Hsp90, triggering the resistance (R) genes that in turn set off the innate immune responses after a pathogen attack [22]. The phosphatase-like protein shares domain similarity with the Hsp90 co-chaperones p23 and Sgt1, providing strong indication that the two proteins detected here, in Trypanosoma brucei, indeed interact, similarly to what occurs in plants and human [22], [23].

### Phylogenetic Analysis

**Figure 4:**
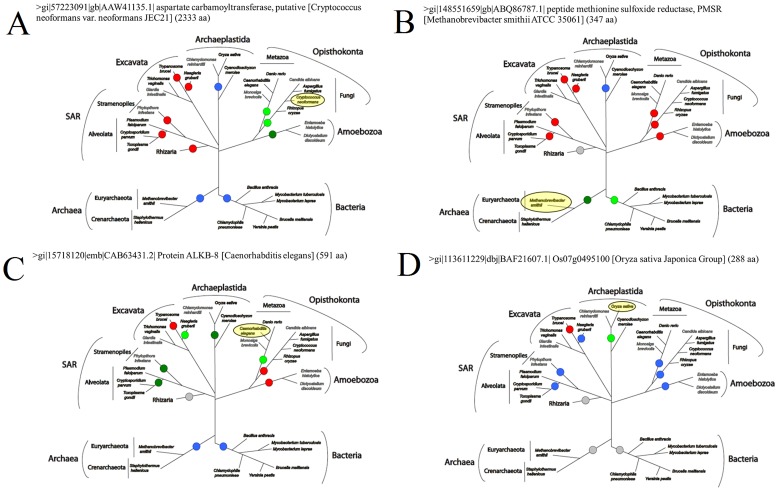
Phylogenetic trees showing examples of the four categories of evolutionary events. These trees show examples of all the gene evolutionary events observed in this study: A) Gene fusion event detected in *Cryptococcus neoformans*; this represents a unique fusion event which most likely happened before the diversification of unikonts. B) Gene fusion event detected in *Methanobrevibacter smithii*; this represents a unique fission event that probably occurred in the *Eukaryotes* superkingdom. C) Gene fusion detected in *Caenorhabditis elegans*; this represents a multiple gene event, including gene fusions and gene fissions. D) Gene fusion detected in *Oryza Sativa*; this was classified as a non-conclusive gene event, as there was not enough sequencing data to support any hypothesis regarding specific gene fusion or fission events. The colored dots along the tree branches represent the state of the protein in each lineage, based on BLAST analysis. *Red*: the protein pair is separate (two different proteins), *Green*: the protein pair is fused, *Blue*: only one part of the fused protein is conserved, either the first or the second member of the protein pair, *Grey*: Absence of either proteins, or not enough data to conclude the presence of the protein pair. The highlighted oval shape indicates the species in which the fusion protein was identified. For a full phylogenetic profile of every result in this study, please see Table S6.

All identified fused protein pairs that passed the backward BLAST verification step were also analyzed to determine their evolutionary history (Table 2). The state of each protein pair (fused or separate) in the major organism lineages, was mapped onto a schematic phylogenetic tree, showing the relationships between these organism groups. From this, conclusions could be drawn about when the fusion or fission event took place, and whether a unique event or multiple fusions/fissions have occurred throughout the course of evolution. This phylogenetic profiling, led to the classification of the fusion events into four major categories: Unique gene fusion events (Figure 4A), Unique gene fission events (Figure 4B), Multiple gene fusion/fission events (4C), and non-conclusive gene evolutionary pattern (Figure 4D). For the unique fusion and fission events, these can be further classified based on the lineage in which the event took place (Table S6). Of the 16 unique fusion events, three occurred in unikonts, four in metazoa, seven in fungi, one in plants, and one in bacteria (Table S6). Of the 10 unique fission events, five occurred in *T. brucei*, three in excavates, and two in eukaryotes (Table S6). Nine multiple fusion/fission and two non-conclusive events were also detected (Table S6). In addition, another seven putative unique fission events in *T. brucei*, one unique fusion event in red algae, as well as two putative multiple fusion/fission events were detected (Table S6). This analysis also allowed us to focus on the results that appear fused in the host organism (*Homo sapiens*). We found that almost 43% of the verified results (21 out of 49) are found fused in the *Homo sapiens* genome while they are encoded by separate genes in *T. brucei* (see Table S2, S3, and S4). Such protein-protein interactions which are specific to the parasite but not the host, comprise good drug target candidates. Theoretically, if the interaction is crucial to the survival or growth of *T. brucei,* designing a specific inhibitor for such an interaction would result in specific inhibition of the parasite’s growth, without adversely affecting the host. Such an approach has already been proposed for the heterodimeric DNA topoisomerase IB enzyme of *T. brucei* [24], [25]. Structural information is available for homologs of most of the domains that participate in the gene fusion events identified here (Table S7), and this information can be used in molecular modelling studies to further explore the potential protein-protein interactions, and to design specific inhibitors which block such interactions, as potential drugs to combat trypanosomiasis.

**Table 2:** Evolutionary categories of the fusion events detected in this study.

Possible (37)			Putative (10)		
Gene fusion	Gene fission	Multiple event	Gene fusion	Gene fission	Multiple event
Fungi (7)	T. brucei (5)	(9)	Red Algae (1)	T. brucei (7)	(2)
Metazoa (4)	Excavata (3)				
Unikonts (3)	Eukaryotes (2)				
Alveolata (2)					
Plants (1)					
Bacteria (1)					

After the phylogenetic analysis of each protein result, it was possible to classify some of the events according to each protein pair’s evolution throughout the tree of life. The categorization here was done by obtaining the evolutionary pattern from each event’s history from the respective tree of life, for example, four fusion events were detected in the *Metazoa* lineage, three fission events probably occurred in the *Excavata*, etc. For more details please refer to the text, and Table S6.

### Conclusions

The present analysis was aiming to identify novel protein-protein interactions through the use of the gene fusion analysis method in *Trypanosoma brucei*. Several studies using this technique have been published, but most have focused on bacteria and fungi [9], [12], [26], [27], [28]. A preliminary analysis for *T. brucei* included only a small number of organisms [5], whereas in this study, we chose organisms so that they would represent every major lineage of the tree of life. In total, 19 organisms were used for the detection of fusion events in the complete genome of *Trypanosoma brucei*.

After the analysis, 49 results were identified and confirmed through the best reciprocal BLAST hit test, and thus represent potential protein-protein interactions. The results were then subjected to extensive search through the KEGG and CDD databases to extract relevant biological information of the proteins concerned.

Evolutionary analysis of the fusion events shows that such fusion and fission events are not confined to a certain kingdom, but are found in nearly all organism families. Fission events are quite common in *T. brucei*, although this may be due to a bias of the method towards the organism used as a reference.

Based on Gene Ontology annotation, approximately 40% of our results have unknown biological process, 9% have unknown molecular function, and 59% unknown cellular component. Importantly, 13% of the PPIs detected by this analysis have already been reported to interact, based on experimental data (e.g. [51]), which demonstrates the credibility of the domain fusion analysis method. The most medically important candidates are the 43% of the results that were found as separate proteins in *T. brucei*, and fused in the human genome. Inhibition of these parasite-specific protein-protein interactions could thus serve as promising drug targets with possibly few or no side-effects.

## Supporting Information

Table S1 **The initial unique results found after the analysis performed using the SAFE software.** Each code given below represents the GI number for each fused protein in each organism, according to the FASTA files used for the analysis, which are shown in Table 1. Codes highlighted in yellow are the fusion events that were successfully verified with backward BLAST, and which are discussed in more detail in the text and Tables S2, S3, and S4.
(PDF)

Table S2 **Fusion events detected in this study, for which functional annotation is available.** This table includes all the protein pairs that were found to participate in fusion events through the automated analysis using the SAFE software and verified by backward BLAST, and for which functional annotation is available for both proteins. The results are grouped by organism (first column) and the common fusion events between the organisms are marked with a distinct color (e.g. red, yellow, cyan, etc.) in the second column. A description of each protein that is involved in the fusion event is also shown, along with the ORF numbers, and the Protein IDs. The table also contains information from the BLAST analysis, displaying the percentage of identities (common amino acid residues in the sequences compared), and the e-value of each result. In the Description column, there is a short description of each event. The description is highlighted in yellow when the two proteins have been previously reported to interact or co-exist in a protein complex, with the respective references shown; the symbol [p] designates participation in the same biological pathway. Finally, the last column displays information about the fate of the protein pair in *Homo sapiens*: f: the protein pair is fused, s: the protein pair is separate (two different proteins), a/b: only one part of the fused protein is conserved in humans, either the first (a) or the second (b), f/s: the protein pair is found in both fused and separate configurations.
(PDF)

Table S3 **Fusion events detected in this study, for which no functional annotation is available.** This table includes all the protein pairs that were found to participate in fusion events through the automated analysis using the SAFE software and verified by backward BLAST, and for which no functional annotation is available for either protein, i.e. both are designated as “hypothetical”. Data are shown/marked as described in the legend for Table S2. In the description column, some data from the Conserved Domains Database (CDD) is presented, mainly by annotations using inference.
(PDF)

Table S4 **Fusion events detected in this study, for which only partial functional annotation is available.** This table includes all the protein pairs that were found to participate in fusion events through the automated analysis using the SAFE software and verified by backward BLAST, and for which functional annotation is only available for one of the two proteins, the other being designated as “hypothetical”. Such results identify novel interactions, and a protein function can be attributed to the hypothetical proteins through the careful in-depth research of each fusion event. Data are shown/marked as described in the legend for Table S2.
(PDF)

Table S5 **Gene ontology (GO) annotation of the fusion events identified.** The Uniprot gene ontology (GO) annotations (biological process, molecular function, cellular component), as well as the Conserved Domains Database (CDD) annotations, are shown for the 49 results that passed the backward BLAST verification; as some of the events were found multiple times, the common events were reduced to one before the GO analysis, resulting in 39 unique protein pairs. No significant bias is apparent for the biological process of the proteins involved in the fusion events in general, nor for their respective molecular function. Based on the GO annotations, approximately 40% of our results had unknown biological process (compared to ∼71% for the whole genome), 9% of our results had unknown molecular function (compared to ∼39% for the whole genome), and 59% of our results had unknown cellular component (compared to ∼47% for the whole genome).
(XLSX)

Table S6 **Phylogenetic trees on which the evolutionary categorization of the fusion events was based.** These trees show the evolution of each protein pair throughout the tree of life. The highlighted oval shape indicates the species in which the fusion protein was identified. The colored dots along the tree branches represent the state of the protein in each lineage, based on BLAST analysis. *Red*: the protein pair is separate (two different proteins), *Green*: the protein pair is fused, *Blue*: only one part of the fused protein is conserved, either the first or the second member of the protein pair, *Grey*: Absence of both proteins, or not enough data to conclude the presence of the protein pair.
(PDF)

Table S7 **Available structural information for homologs of the domains which participate in the gene fusion events identified.** For each of the 49 fusion events verified by reverse BLAST, the accession numbers of the corresponding protein pair in *T. brucei* is given. To identify available structural information, protein BLAST was used to compare each protein sequence against the protein sequences extracted from the PDB three-dimensional structure records. For each protein, the accession number for the top matching PDB record is given, as well as details of the percent identity and the residue range for the match. The last column gives the residue range for the part of each protein that participates in the fusion event, as identified initially by the SAFE software, which shows that in most cases, the PDB hit largely overlaps with the fusion domain. The available structural information for the proteins that participate in fusions events can be used in molecular modelling studies to further explore the potential protein-protein interactions, and to design specific inhibitors which block such interactions, as potential drugs to combat trypanosomiasis.
(PDF)
