# Supplementary material for: Gene Fusion Analysis in the Battle against the African Endemic Sleeping Sickness
Source: PLoS One. 2013 Jul 17;8(7):e68854. doi: 10.1371/journal.pone.0068854 (PMC3714255; doi:10.1371/journal.pone.0068854)
Supplement: Table S1 — The initial unique results found after the analysis performed using the SAFE software. Each code given below represents the GI number for each fused protein in each organism, according to the FASTA files used for the analysis, which are shown in Table 1. Codes highlighted in yellow are the fusion events that were successfully verified with backward BLAST, and which are discussed in more detail in the text and Tables S2, S3, and S4. (PDF) [file pone.0068854.s001.pdf]

Trichomonas vaginalis	*Mycobacterium tuberculosis*	*Mycobacterium leprae*	*Yersinia pestis*	*Bacillus anthracis*	*Brucella melitensis*	*Chlamydophila pleumoniae*	*Methanobrevibacter smithii*	*Staphylothermus hellenicus*	*Plasmodium falciparum*	*Cryptosporidium parvum*	*Toxoplasma gondii*	*Oryza sativa*	*Cyanidioschyzon merolae*	*Caenorhabditis elegans*	*Danio rerio*	*Cryptococcus neoformans*	*Rhizopus oryzae*	*Aspergillus fumigatus*
121917718	13883346	**13094041**	21960990	49182220	17985243	**7189996**	148552575	297256480	255528889	32399059	TGME49_110860	255671004	CMF058C	313569965	**Q5XJ54**	57230869	**RO3T_01042**	**Afua_1g10830**
121917063	13883207	13093327	**21960686**	**49182202**	**17983784**		148552575	297255526	254832632	32398937	TGME49_111240	255670109	CMH019C	227452788	**Q08C92**	57230191	RO3T_01177	**Afua_1g11540**
121916965	13882255	13092904	21960013	49181488	17982999		**148551659**	**297255006**	23615364	32398644	TGME49_060310	**113631616**	CMI098C	194474366	Q6TGX5	57228183	RO3T_01367	Afua_2g13980
121911850	13881260	13092853	21959982	49181291	17982824				23504716		TGME49_074060	113623983	**CMM263C**	169404869	**Q7ZW29**	**57226667**	**RO3T_02488**	**Afua_3g03970**
121910829			21958563	49181213					23499081		TGME49_023450	113611299	CMO072C	92110144	**Q8JHH7**	57223222	**RO3T_04092**	Afua_4g02660
121908580			21957774	**49181135**					**23497583**		TGME49_024190	**113611229**	**CMO271C**	89179570	Q5XJU0	**57223091**	**RO3T_05874**	Afua_5g06510
121905239				49178899					23496781		TGME49_089780	113578235	**CMQ255C**	73912806	E4VNZ2	57223075	**RO3T_06091**	Afua_5g13520
121900500				49178758					23494932		**TGME49_091930**	113578178	**CMT489C**	47270748	Q4VBW1	57222950	RO3T_07528	Afua_6g02070
121900173				49177103							TGME49_067550	113564811		27674035	**B0S700**		**RO3T_09902**	Afua_6g07980
121900061											**TGME49_034510**	113564653		16604139	Q5SPR9		**RO3T_11245**	**Afua_6g11310**
121898436											TGME49_019550	113548402		**15718120**	Q4V9K8		RO3T_11669	Afua_7g00520
121897135											TGME49_081430	113536949		15150693	A2BGP4		RO3T_11697	Afua_7g08480
121882335											TGME49_020140	113536214		**14625283**	B8JJN8		RO3T_15620	
												113535551		**14573988**	Q804C3		**RO3T_16834**	
												56784314		13384544	A3KP83			
												54290194		**7332076**	**Q1ED17**			
												15623934		**3881810**	B3DKQ3			
												**14209584**		3879646	Q6NV13			
														3879486	Q6PH26			
														3875822	**Q7T3F6**			
														2394478	Q5RH81			
														**2315645**	**Q6DBR7**			
														**2291243**	**A8E528**			
														2291231				
														**1280169**				
														861246				
