# Supplementary material for: Gene Fusion Analysis in the Battle against the African Endemic Sleeping Sickness
Source: PLoS One. 2013 Jul 17;8(7):e68854. doi: 10.1371/journal.pone.0068854 (PMC3714255; doi:10.1371/journal.pone.0068854)
Supplement: Table S2 — Fusion events detected in this study, for which functional annotation is available. This table includes all the protein pairs that were found to participate in fusion events through the automated analysis using the SAFE software and verified by backward BLAST, and for which functional annotation is available for both proteins. The results are grouped by organism (first column) and the common fusion events between the organisms are marked with a distinct color (e.g. red, yellow, cyan, etc.) in the second column. A description of each protein that is involved in the fusion event is also shown, along with the ORF numbers, and the Protein IDs. The table also contains information from the BLAST analysis, displaying the percentage of identities (common amino acid residues in the sequences compared), and the e-value of each result. In the Description column, there is a short description of each event. The description is highlighted in yellow when the two proteins have been previously reported to interact or co-exist in a protein complex, with the respective references shown; the symbol [p] designates participation in the same biological pathway. Finally, the last column displays information about the fate of the protein pair in Homo sapiens: f: the protein pair is fused, s: the protein pair is separate (two different proteins), a/b: only one part of the fused protein is conserved in humans, either the first (a) or the second (b), f/s: the protein pair is found in both fused and separate configurations. (PDF) [file pone.0068854.s002.pdf]

Organism	Fusion event	Protein ID	% Identities	Fusion e-value	Open Reading Frame	Protein name	Description	Gene in *Homo Sapiens*
Mycobacterium leprae	13094041	XP_001218898.1<br>XP_829571.1	30<br>30	3e-005<br>1e-010	Tb927.1.1960<br>Tb11.01.6780	chaperone protein DNAJ<br>chaperone protein DnaJ	chaperone [p]	f/s
Yersinia pestis	21960686	XP_001218898.1<br>XP_823081.1	30<br>31	2e-007<br>2e-013	Tb927.1.1960<br>Tb10.6k15.2000	chaperone protein DNAJ<br>chaperone protein DnaJ	chaperone [p]	f/s
Bacillus anthracis	49181135	XP_845692.1<br>XP_823081.1	31<br>29	2e-009<br>2e-011	Tb927.7.680<br>Tb10.6k15.2000	chaperone protein DNAJ<br>chaperone protein DnaJ	chaperone [p]	f/s
Brucella melitensis	17983784	XP_822465.1<br>XP_828437.1	28<br>30	3e-044<br>1e-004	Tb10.70.5650/ Tb10.70.5670<br>Tb11.02.1040	elongation factor 1-alpha<br>XPA-interacting protein	-	s
Chlamydophila pleumoniae	7189996	XP_847169.1<br>XP_826981.1	28<br>28	9e-008<br>6e-006	Tb927.8.3380<br>Tb09.160.4380	electron transfer protein<br>succinate dehydrogenase	parts of protein complex [p] [5, 51]	f
Staphylothermus hellenicus	297255006	XP_822277.1<br>XP_844534.1	27<br>29	0.006<br>7e-019	Tb10.100.0060<br>Tb927.4.3840	centromere/microtubule binding protein<br>nucleolar protein	proposed coexistence/ cooperation [p]	f/b
Plasmodium falciparum	23497583	XP_828707.1<br>XP_822502.1	27<br>33	0.001<br>6e-083	Tb11.02.4200<br>Tb10.70.5200	6-phosphogluconolactonase<br>glucose-6-phosphate 1-dehydrogenase	unique enzyme in P.falciparum [p] [14]	f
Oryza sativa	113631616	XP_823399.1<br>XP_823179.1	27<br>36	6e-007<br>1e-055	Tb10.389.1850<br>Tb10.6k15.0820	NAD or NADP dependent oxidoreductase<br>oxidoreductase	proposed parts of protein complex [p]	s
Cyanidioschyzon merolae	CMM263C	XP_844283.1<br>XP_827055.1	49<br>41	1e-120<br>2e-012	Tb927.4.1330<br>Tb09.160.5070	DNA topoisomerase IB, large subunit<br>DNA topoisomerase type IB small subunit	parts of protein complex [p] [5, 24, 25]	f/s
	CMO271C	XP_828209.1<br>XP_828072.1	27<br>28	2e-006<br>1e-004	Tb11.47.0031<br>Tb10.61.0250	cell-division control protein 2 homolog 6<br>mitogen-activated protein kinase	share homology [p]	f/s
	CMQ255C	XP_845050.1<br>XP_845052.1	48<br>46	0.0<br>4e-071	Tb927.5.3800<br>Tb927.5.3820	glutamine hydrolysing (not ammonia-dependent) carbomoyl phosphate synthase<br>aspartate carbamoyltransferase	interacting proteins [p] [5]	f/s
	CMT489C	XP_843903.1<br>XP_845469.1	34<br>31	3e-005<br>3e-046	Tb927.3.3100<br>Tb927.6.3510	peptidyl-prolyl cis-trans isomerase NIMA-interacting 4<br>tRNA modification enzyme	-	s/b
Caenorhabditis elegans	14573988	XP_844668.1<br>XP_846985.1	33<br>34	2e-004<br>1e-055	Tb927.4.5200<br>Tb927.8.1510	nucleoporin (NUP54/57)<br>ATP-dependent DEAD/H RNA helicase	paralog result [p]	b
	2315645	XP_827599.1<br>XP_951693.1	35<br>29	9e-006<br>3e-012	Tb09.211.4570<br>Tb927.2.5210	short-chain dehydrogenase<br>3-oxoacyl-(ACP) reductase	SDR protein family [p]	f/s
	2291243	XP_823206.1<br>XP_823081.1	29<br>38	9e-015<br>1e-013	Tb10.6k15.0460<br>Tb10.6k15.2000	chaperone protein DnaJ<br>chaperone protein DnaJ	chaperone [p]	f/s
	1280169	XP_843940.1<br>XP_845045.1	36<br>28	1e-012<br>0.004	Tb927.3.3480<br>Tb927.5.3750	U2 small nuclear ribonucleoprotein B<br>RNA-binding protein	RNA-containing proteins	f/s

Danio rerio	Q5XJ54	XP_844769.1<br>XP_803662.1	33<br>41	1e-033<br>1e-018	Tb927.5.950<br>Tb09.160.2210	thioredoxin-like protein<br>glutaredoxin-like protein	Homologous [p]	f/s
	Q08C92	XP_844345.1<br>XP_803801.1	27<br>46	3e-007<br>3e-104	Tb927.4.1950<br>Tb09.160.3520	NADPH--cytochrome p450 reductase<br>radical SAM domain protein	-	f/s
	Q8JHH7	XP_829376.1<br>XP_845565.1	30<br>55	5e-009<br>9e-038	Tb11.01.4750<br>Tb927.6.4480	elongation factor 1 gamma<br>valyl-tRNA synthetase	same pathway [p]	f/s
	Q1ED17	XP_844283.1<br>XP_827055.1	46<br>33	5e-113<br>2e-010	Tb927.4.1330<br>Tb09.160.5070	DNA topoisomerase IB, large subunit<br>DNA topoisomerase type IB small subunit	parts of protein complex [p] [5, 24, 25]	f/s
Cryptococcus neoformans	57226667	XP_829573.1<br>XP_951511.1	27<br>46	6e-010<br>1e-106	Tb11.01.6800<br>Tb927.2.1780	1-acyl-sn-glycerol-3-phosphate acyltransferase protein<br>N-acetylglucosaminyl-phosphatidylinositol biosynthetic protein	-	s
	57223091	XP_845050.1<br>XP_845052.1	47<br>45	0.0<br>4e-073	Tb927.5.3800<br>Tb927.5.3820	glutamine hydrolysing (not ammonia-dependent) carbomoyl phosphate synthase<br>aspartate carbamoyltransferase	interacting proteins [p] [5]	f/s
Rhizopus oryzae	RO3T_05874	XP_827933.1<br>XP_822660.1	37<br>43	2e-045<br>6e-016	Tb10.61.2180<br>Tb10.70.3160	proteasome regulatory non-ATPase subunit 8<br>60S ribosomal protein L30	-	s
	RO3T_06091	XP_845050.1<br>XP_845052.1	49<br>46	0.0<br>6e-076	Tb927.5.3800<br>Tb927.5.3820	glutamine hydrolysing (not ammonia-dependent) carbomoyl phosphate synthase<br>aspartate carbamoyltransferase	interacting proteins [p] [5]	f/s
	RO3T_11245 (RO3G_11246)	XP_823179.1<br>XP_845051.1	37<br>30	3e-048<br>9e-014	Tb10.6k15.0820<br>Tb927.5.3810	oxidoreductase<br>orotidine-5-phosphate decarboxylase/orotate phosphoribosyltransferase	-	s
Aspergillus fumigatus	Afua_1g10830	XP_843817.1<br>XP_822976.1	47<br>38	8e-062<br>4e-041	Tb927.3.2230<br>Tb10.6k15.3250	succinyl-CoA synthetase alpha subunit<br>succinyl-CoA ligase [GDP-forming] beta-chain	parts of protein complex [p]	s
	Afua_3g03970	XP_828738.1<br>XP_828456.1	42<br>37	2e-016<br>9e-039	Tb11.02.4485<br>Tb11.02.1230	cytochrome b5<br>NADH-cytochrome b5 reductase	same reaction [p]	f/s
	Afua_6g11310	XP_845050.1<br>XP_845052.1	49<br>47	0.0<br>2e-070	Tb927.5.3800<br>Tb927.5.3820	glutamine hydrolysing (not ammonia-dependent) carbomoyl phosphate synthase<br>aspartate carbamoyltransferase	interacting proteins [p] [5]	f/s
