# Supplementary material for: Gene Fusion Analysis in the Battle against the African Endemic Sleeping Sickness
Source: PLoS One. 2013 Jul 17;8(7):e68854. doi: 10.1371/journal.pone.0068854 (PMC3714255; doi:10.1371/journal.pone.0068854)
Supplement: Table S3 — Fusion events detected in this study, for which no functional annotation is available. This table includes all the protein pairs that were found to participate in fusion events through the automated analysis using the SAFE software and verified by backward BLAST, and for which no functional annotation is available for either protein, i.e. both are designated as “hypothetical”. Data are shown/marked as described in the legend for Table S2. In the description column, some data from the Conserved Domains Database (CDD) is presented, mainly by annotations using inference. (PDF) [file pone.0068854.s003.pdf]

Organism	Fusion event	Protein ID	% Identities	Fusion e-value	Open Reading Frame	Protein name	Description	Gene in *Homo Sapiens*
Toxoplasma gondii	TGME49_034510	XP_846297.1	36	1e-008	Tb927.7.6770	hypothetical protein	Acyl CoA binding protein, Ankyrin repeats (CDD)	f/b
		XP_827785.1	27	1e-006	Tb10.389.0520	hypothetical protein		
Caenorhabditis elegans	15718120	XP_845777.1	30	1e-013	Tb927.7.1530	hypothetical protein	SAM domains, 2OG-Fe(II) oxygenase superfamily, contains RRM motifs (CDD)	f/s
		XP_822990.1	30	1e-025	Tb10.6k15.3090	hypothetical protein		
	14625283	XP_829015.1	30	2e-009	Tb11.01.1270	hypothetical protein	DEAD-box helicase, zinc finger (CDD)	b
		XP_828952.1	33	4e-052	Tb11.01.0610	hypothetical protein		
Danio rerio	Q7T3F6	XP_829326.1	27	0.002	Tb11.01.4480	hypothetical protein	Phafin domain, FYVE zinc finger domain (CDD)	f/s
		XP_827443.1	32	3e-012	Tb09.211.2990	hypothetical protein		
	A8E528	XP_822480.1	30	0.005	Tb10.70.5470	hypothetical protein	Ubiquitin like protein, with NLI interacting factors (CDD)	f/s
		XP_826990.1	27	4e-007	Tb09.160.4460	hypothetical protein		
Rhizopus oryzae	RO3T_01042 (RO3G_01043)	XP_846297.1	40	3e-008	Tb927.7.6770	hypothetical protein	Acyl CoA binding protein (CDD)	f/s
		XP_828884.1	27	2e-004	Tb11.01.0440	hypothetical protein		
