# Supplementary material for: Gene Fusion Analysis in the Battle against the African Endemic Sleeping Sickness
Source: PLoS One. 2013 Jul 17;8(7):e68854. doi: 10.1371/journal.pone.0068854 (PMC3714255; doi:10.1371/journal.pone.0068854)
Supplement: Table S4 — Fusion events detected in this study, for which only partial functional annotation is available. This table includes all the protein pairs that were found to participate in fusion events through the automated analysis using the SAFE software and verified by backward BLAST, and for which functional annotation is only available for one of the two proteins, the other being designated as “hypothetical”. Such results identify novel interactions, and a protein function can be attributed to the hypothetical proteins through the careful in-depth research of each fusion event. Data are shown/marked as described in the legend for Table S2. (PDF) [file pone.0068854.s004.pdf]

Organism	Fusion event	Protein ID	% Identities	Fusion e-value	Open Reading Frame	Protein name	Description	Gene in *Homo Sapiens*
Bacillus anthracis	49182202	XP_846892.1	38	3e-023	Tb927.8.550	peptide methionine sulfoxide reductase	Discussed in manuscript	s
		XP_829255.1	37	5e-013	Tb11.01.3760	hypothetical protein		
Methanobrevibacter smithii	148551659	XP_846892.1	38	5e-026	Tb927.8.550	peptide methionine sulfoxide reductase	Discussed in manuscript	s
		XP_829255.1	34	5e-011	Tb11.01.3760	hypothetical protein		
Toxoplasma gondii	TGME49_091930	XP_827326.1	31	3e-005	Tb09.211.1800	hypothetical protein	Domains: RNA recognition motif	b
		XP_845376.1	28	8e-005	Tb927.6.2550	RNA-binding protein		
Oryza sativa	113611229	XP_844119.1	36	0.008	Tb927.3.5290	hypothetical protein	Discussed in manuscript	f
		XP_829146.1	57	4e-067	Tb11.01.2590	protein kinase ck2 regulatory subunit		
	14209584	XP_845265.1	27	3e-006	Tb927.6.1430	hypothetical protein	Domains: Peptidase C19	f/a
		XP_827631.1	27	1e-073	Tb09.211.4910	ubiquitin carboxyl-terminal hydrolase		
Caenorhabditis elegans	7332076	XP_846183.1	27	3e-004	Tb927.7.5600	hypothetical protein	Domains: phenylalanyl-tRNA synthetase	f/s
		XP_828320.1	28	5e-012	Tb11.22.0005	phenylalanyl-tRNA synthetase		
	3881810	XP_846301.1	32	0.009	Tb927.7.6810	hypothetical protein	Domains: EF-hand, calcium binding motif	f/a/b
		XP_823096.1	34	2e-005	Tb10.6k15.1830	centrin		
Danio rerio	Q7ZW29	XP_828351.1	27	0.001	Tb11.02.0280	hypothetical protein	Domains: Zinc finger	f/s
		XP_846079.1	32	3e-045	Tb927.7.4560	histone acetyltransferase		
	B0S700	XP_822515.1	28	9e-006	Tb10.70.5050	hypothetical protein	Domains: Protein tyrosine phosphatase-like protein, PTPLA	f/s
		XP_823281.1	32	2e-020	Tb10.406.0290	protein tyrosine phosphatase		
	Q6DBR7	XP_001219015.1	31	1e-022	Tb927.1.3170	hypothetical protein	Discussed in manuscript	f/s
		XP_001219018.1	29	0.001	Tb927.1.3200	phosphatase-like protein		
Rhizopus oryzae	RO3T_02488	XP_829472.1	38	5e-031	Tb11.01.5750	hypothetical protein	Domains: XPG N/I regions	s
		XP_843679.1	41	5e-071	Tb927.3.830	flap endonuclease-1 (FEN-1)		
	RO3T_04092	XP_847085.1	48	8e-144	Tb927.8.2520	acetyl-CoA synthetase	Domains: AMP-binding, NTPase/HAM1 family	s
	(RO3G_04093)	XP_823221.1	48	2e-044	Tb10.6k15.0290	hypothetical protein		
	RO3T_09902	XP_951738.1	50	0.0	Tb927.2.5980	ATP-dependent Clp protease subunit, heat shock protein 100 (HSP100)	Domains: ATPases associated with diverse cellular activities	s
		XP_844286.1	30	4e-026	Tb927.4.1360	hypothetical protein		
	RO3T_16834	XP_001219015.1	34	1e-025	Tb927.1.3170	hypothetical protein	Discussed in manuscript	f/s
		XP_001219018.1	28	1e-004	Tb927.1.3200	phosphatase-like protein		
Aspergillus fumigatus	Afua_1g11540	XP_001219015.1	33	2e-020	Tb927.1.3170	hypothetical protein	Discussed in manuscript	f/s
		XP_001219018.1	27	5e-005	Tb927.1.3200	phosphatase-like protein		
