# Supplementary material for: Gene Fusion Analysis in the Battle against the African Endemic Sleeping Sickness
Source: PLoS One. 2013 Jul 17;8(7):e68854. doi: 10.1371/journal.pone.0068854 (PMC3714255; doi:10.1371/journal.pone.0068854)

# Clear (37)

## Fusion in *Unikonts* (3)

>RO3T_16834 | RO3G_16835 | Rhizopus oryzae hypothetical protein (340 aa)

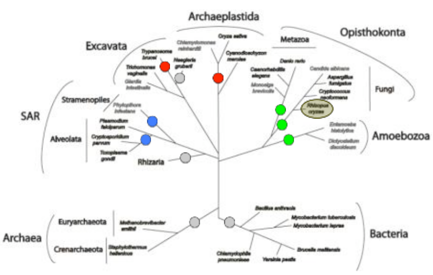


>Afu1g11540 | Aspergillus fumigatus CORD and CS domain protein (315 aa)

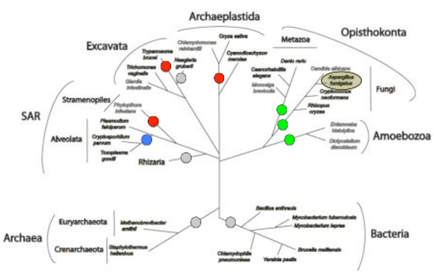


>gi|57223091|gb|AAW41135.1| aspartate carbamoyltransferase, putative [Cryptococcus neoformans var. neoformans JEC21] (2333 aa)

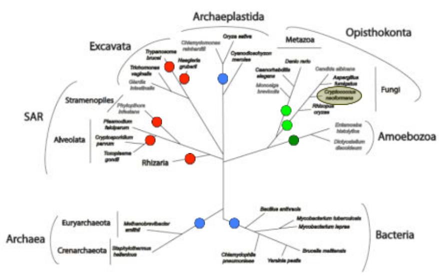


## Fusion in *Metazoa* (4)

>tr|Q7ZW29|Q7ZW29_DANRE MYST histone acetyltransferase 2 OS=Danio rerio (568 aa)

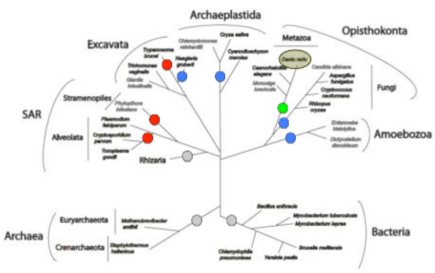


>tr|Q8JHH7|Q8JHH7_DANRE Valyl-tRNA synthetase (Fragment) OS=Danio rerio (425 aa)

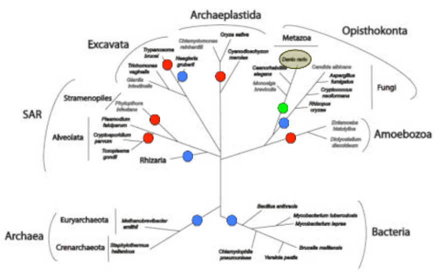


>tr|B0S700|B0S700_DANRE Novel protein (Zgc:63632) OS=Danio rerio (404 aa)

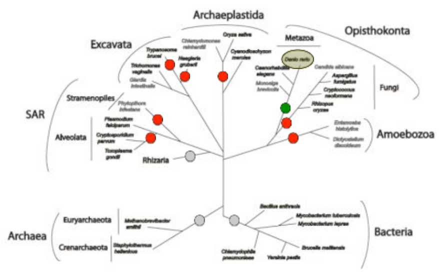


>gi|7332076|gb|AAF60763.1| Hypothetical protein Y54E10A.6 [Caenorhabditis elegans] (507 aa) RECORD REMOVED

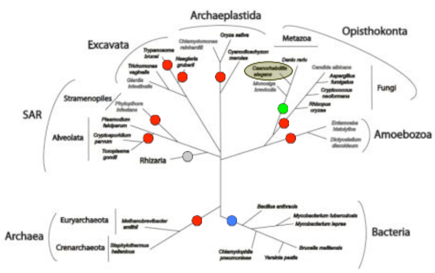

# Fusion in *Alveolata* (2)

>gi|23497583|gb|AAN37124.1| glucose-6-phosphate dehydrogenase-6-phosphogluconolactonase [Plasmodium falciparum 3D7] (910 aa)

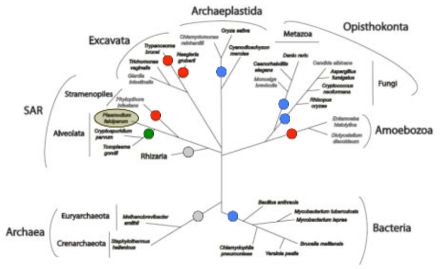


>gi|211966199|gb|EEB01395.1| nucleolar phosphoprotein nucleolin, putative [Toxoplasma gondii ME49] (705 aa)

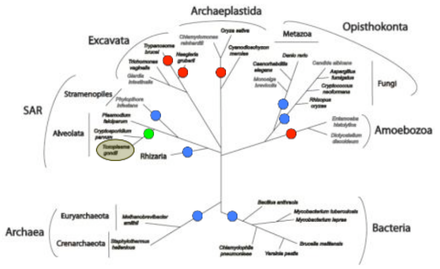


# Fusion in *Fungi* (7)

>gi|57226667|gb|AAW43127.1| transferase, putative [Cryptococcus neoformans var. neoformans JEC21] (783 aa)

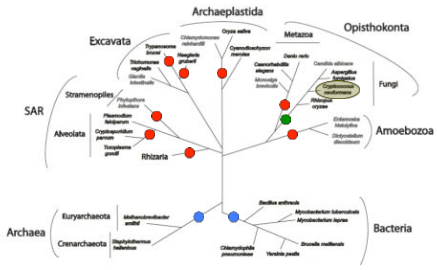


>Afu1g10830 | Aspergillus fumigatus succinyl-CoA synthetase, putative (678 aa)

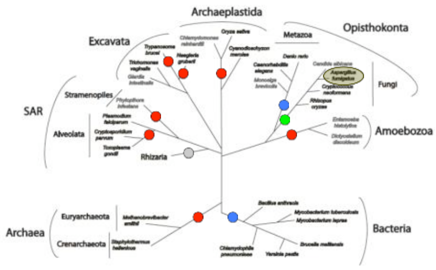


>RO3T_02488 | RO3G_02489 | Rhizopus oryzae hypothetical protein (497 aa)

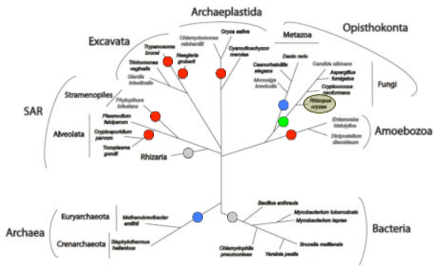


>RO3T_11245 | RO3G_11246 | Rhizopus oryzae orotate phosphoribosyltransferase (515 aa)

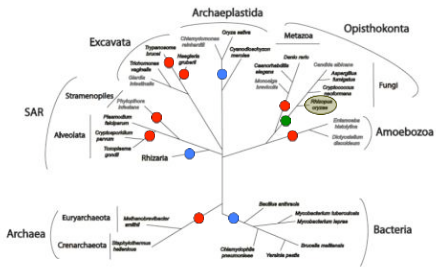


>RO3T_04092 | RO3G_04093 | Rhizopus oryzae hypothetical protein (698 aa)

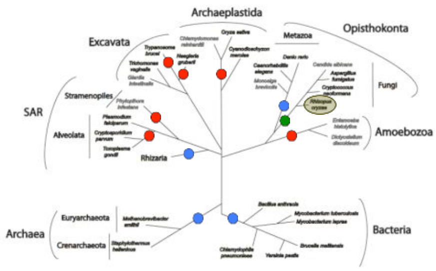


>RO3T_05874 | RO3G_05875 | Rhizopus oryzae hypothetical protein (374 aa)

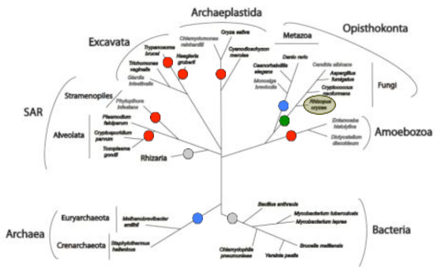


>RO3T_09902 | RO3G_09903 | Rhizopus oryzae hypothetical protein (961 aa)

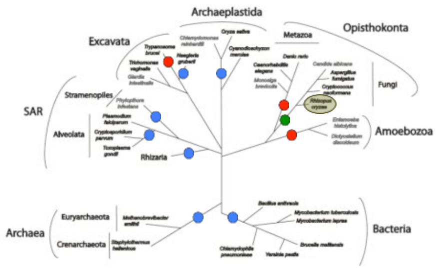

# Fusion in *Plants* (1)

>gi|113631616|dbj|BAF25297.1| Os09g0459800 [Oryza sativa Japonica Group] (634 aa)

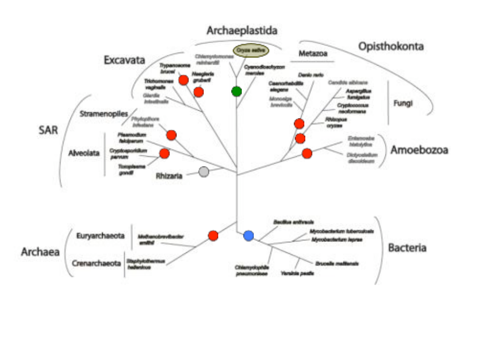


# Fusion in *Bacteria* (1)

>gi|17983784|gb|AAL52935.1| sulfate adenylyltransferase / adenylylsulfate kinase [Brucella melitensis bv. 1 str. 16M] (644 aa)

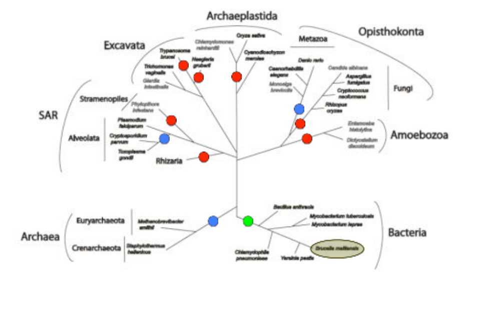


# Fission in *T. brucei* (5)

>gnl|CMER|CMM263C Cyanidioschyzon merolae strain 10D DNA topoisomerase (706 aa)

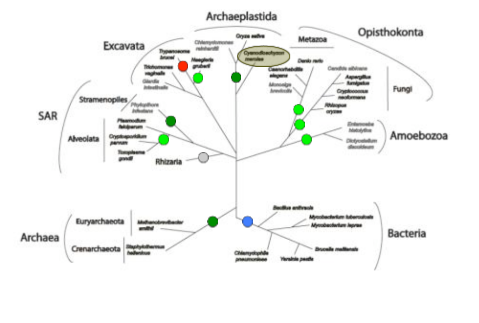


>tr|Q7T3F6|Q7T3F6_DANRE Pleckstrin homology domain containing, family F (With FYVE domain) member 1 OS=Danio rerio (293 aa)

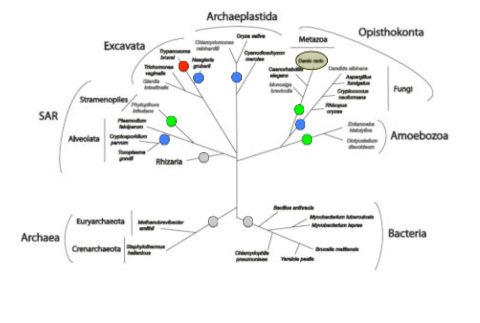


>gi|14209584|dbj|BAB56080.1| putative ubiquitin carboxyl-terminal hydrolase 7 [Oryza sativa Japonica Group] (1108 aa)

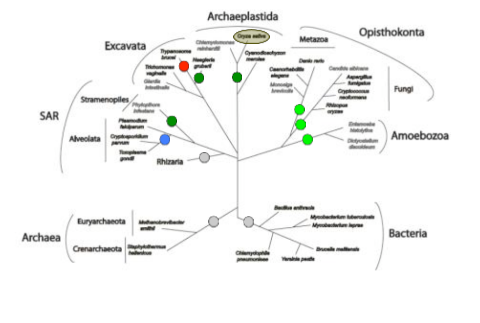


>tr|Q1ED17|Q1ED17_DANRE Topoisomerase (DNA) I, like OS=Danio rerio (758 aa)

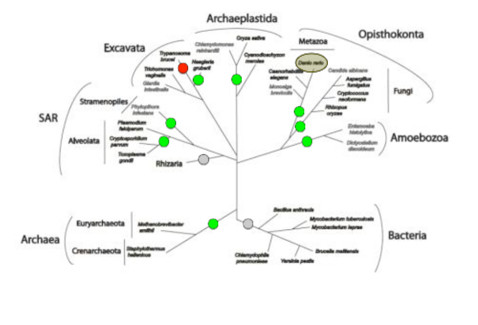


>gi|14573988|gb|AAK68269.1|AC006625_1 Germ line helicase protein 2, confirmed by transcript evidence [Caenorhabditis elegans] (974 aa) RECORD REMOVED

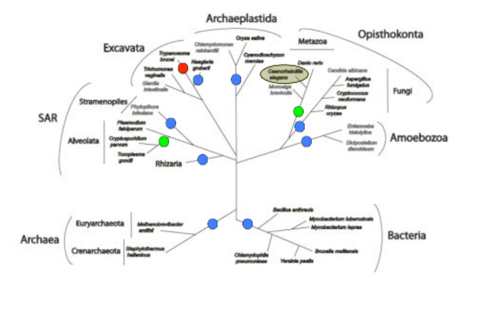

# Fission in *Excavata* (3)

>RO3T_01042 | RO3G_01043 | Rhizopus oryzae predicted protein (213 aa)

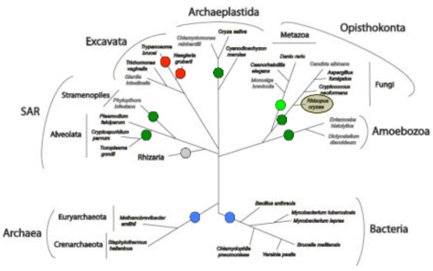


>tr|A8E528|A8E528_DANRE Ublcp1 protein OS=Danio rerio (318 aa)

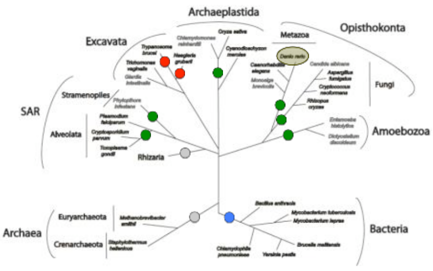


>gi|7189996|gb|AAF38854.1| succinate dehydrogenase, iron-sulfur protein [Chlamydophila pneumoniae AR39] (258 aa)

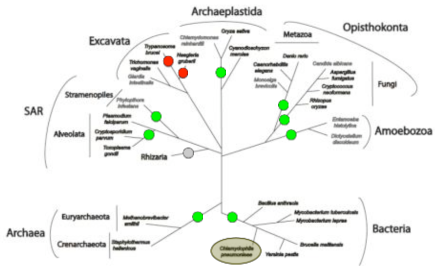


# Fission in *Eukaryotes* (2)

>gi|148551659|gb|ABQ86787.1| peptide methionine sulfoxide reductase, PMSR [Methanobrevibacter smithii ATCC 35061] (347 aa)

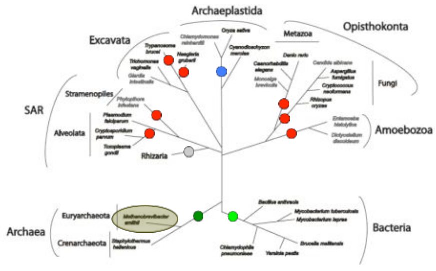


>gi|49182202|gb|AAT57578.1| peptide methionine sulfoxide reductase [Bacillus anthracis str. Sterne] (321 aa)

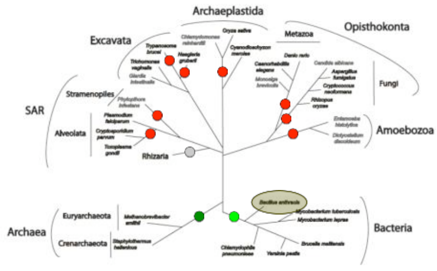


# Multiple fusions/ fissions (9)

>gnl|CMER|CMQ255C Cyanidioschyzon merolae strain 10D CAD complex (carbamoyl-phosphate synthase II / aspartate carbamoyltransferase catalytic chain / dihydroorotase) (2328 aa)

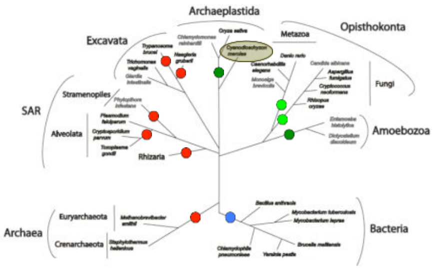


>RO3T_06091 | RO3G_06092 | Rhizopus oryzae hypothetical protein (2273 aa)

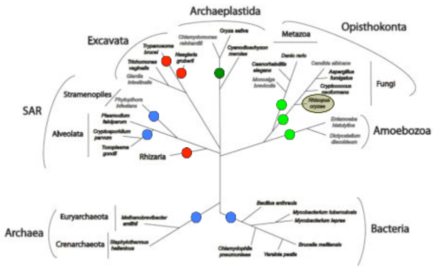


>Afu3g03970 | Aspergillus fumigatus cytochrome-b5 reductase, putative (480 aa)

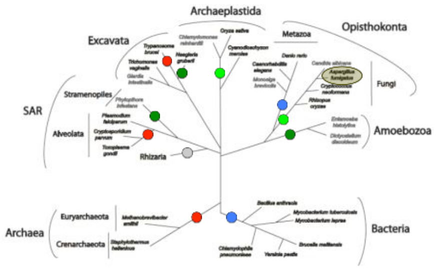


>tr|B6KM18|B6KM18_TOXGO Acyl-CoA-binding protein, putative OS=Toxoplasma gondii (311 aa)

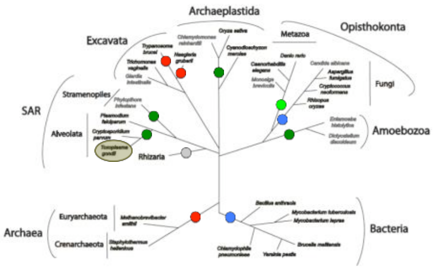


>gi|15718120|emb|CAB63431.2| Protein ALKB-8 [Caenorhabditis elegans] (591 aa)

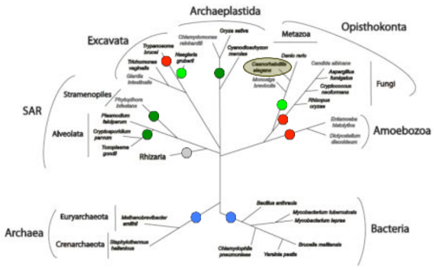


>sp|Q5XJ54|GLRX3_DANRE Glutaredoxin 3 OS=Danio rerio (326 aa)

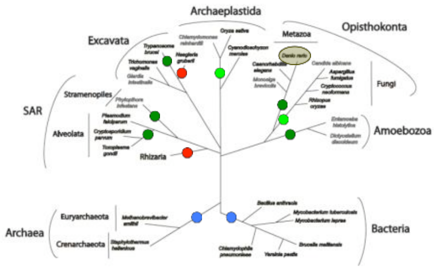

>tr|Q6DBR7|Q6DBR7_DANRE Uncharacterized protein OS=Danio rerio (387 aa)

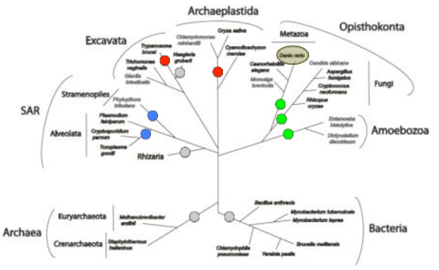


>gi|297255006|gb|ADI31215.1| Fmu (Sun) domain protein [Staphylothermus hellenicus DSM 12710] (383 aa)

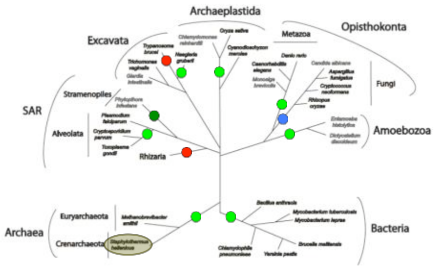


>Afu6g11310 | Aspergillus fumigatus bifunctional pyrimidine biosynthesis protein (PyrABCN), putative (2254 aa)

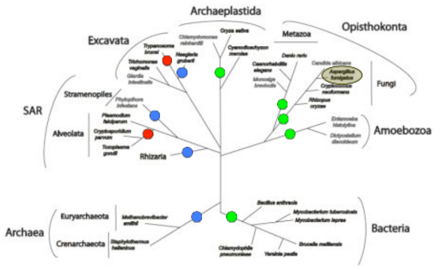


## Undecided (2)

>gi|14625283|gb|AAB52901.2| Germ line helicase protein 1, confirmed by transcript evidence [Caenorhabditis elegans] (763 aa) RECORD REMOVED

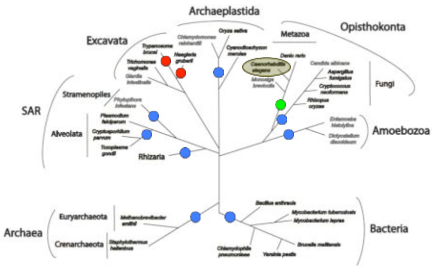


>gi|113611229|dbj|BAF21607.1| Os07g0495100 [Oryza sativa Japonica Group] (288 aa)

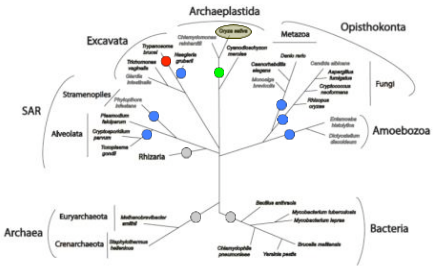


# Putative (10)

## Fission in *T. brucei* (7)

>gi|13094041|emb|CAC32011.1| Hsp70 cofactor [Mycobacterium leprae] (388 aa)

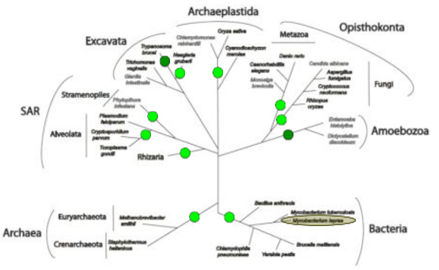


>gi|21960686|gb|AAM87253.1|AE013974_1 chaperone with DnaK; heat shock protein [Yersinia pestis KIM10+] (379 aa)

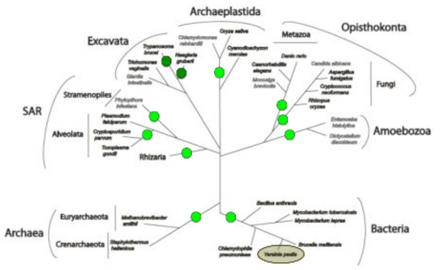


>gi|49181135|gb|AAT56511.1| chaperone protein dnaJ [Bacillus anthracis str. Sterne] (371 aa)

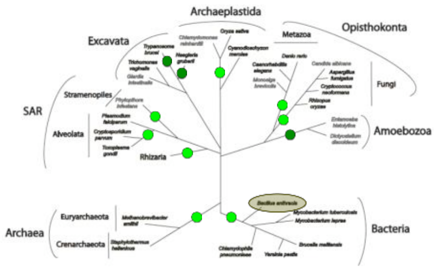


>gi|2315645|gb|AAB66092.1| Dehydrogenases, short chain protein 13 [Caenorhabditis elegans] (257 aa) RECORD REMOVED

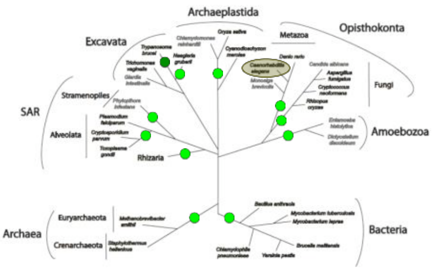


>gi|2291243|gb|AAB65361.1| Dnaj domain (prokaryotic heat shock protein) protein 19, confirmed by transcript evidence [Caenorhabditis elegans] (439 aa) RECORD REMOVED

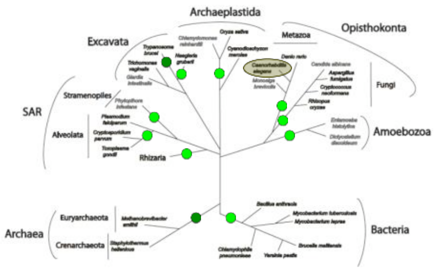


>gi|1280169|gb|AAA98033.1| Rnp (rrm rna binding domain) containing protein 3, confirmed by transcript evidence [Caenorhabditis elegans] (217 aa) RECORD REMOVED

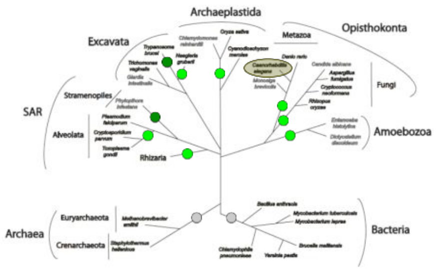

>sp|Q08C92|TYW1_DANRE tRNA wybutosine-synthesizing protein 1 homolog OS=Danio rerio (730 aa)

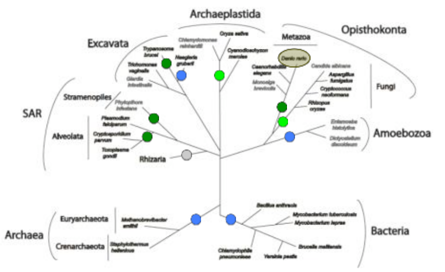


# Fusion in *Red Algae* (1)

>gnl|CMER|CMT489C Cyanidioschyzon merolae strain 10D 2-methylthioadenine synthetase (707 aa)

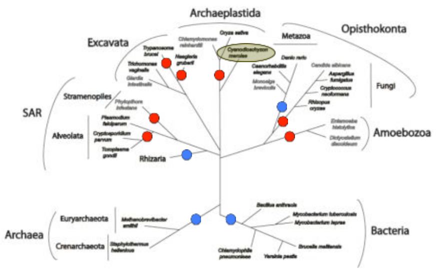


# Multiple fusions/ fissions (2)

>gnl|CMER|CMO271C Cyanidioschyzon merolae strain 10D cell cycle protein kinase CDC7 (456 aa)

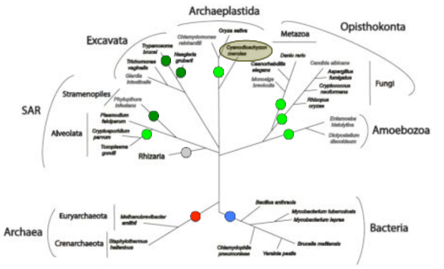


>gi|3881810|emb|CAA94856.1| Protein ZK856.8 [Caenorhabditis elegans] (195 aa)

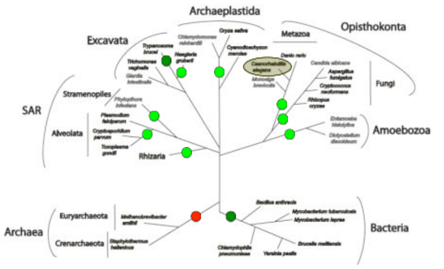

Supplement: Table S6 — Phylogenetic trees on which the evolutionary categorization of the fusion events was based. These trees show the evolution of each protein pair throughout the tree of life. The highlighted oval shape indicates the species in which the fusion protein was identified. The colored dots along the tree branches represent the state of the protein in each lineage, based on BLAST analysis. Red: the protein pair is separate (two different proteins), Green: the protein pair is fused, Blue: only one part of the fused protein is conserved, either the first or the second member of the protein pair, Grey: Absence of both proteins, or not enough data to conclude the presence of the protein pair. (PDF) [file pone.0068854.s006.pdf]
