# Supplementary material for: Gene Fusion Analysis in the Battle against the African Endemic Sleeping Sickness
Source: PLoS One. 2013 Jul 17;8(7):e68854. doi: 10.1371/journal.pone.0068854 (PMC3714255; doi:10.1371/journal.pone.0068854)
Supplement: Table S7 — Available structural information for homologs of the domains which participate in the gene fusion events identified. For each of the 49 fusion events verified by reverse BLAST, the accession numbers of the corresponding protein pair in T. brucei is given. To identify available structural information, protein BLAST was used to compare each protein sequence against the protein sequences extracted from the PDB three-dimensional structure records. For each protein, the accession number for the top matching PDB record is given, as well as details of the percent identity and the residue range for the match. The last column gives the residue range for the part of each protein that participates in the fusion event, as identified initially by the SAFE software, which shows that in most cases, the PDB hit largely overlaps with the fusion domain. The available structural information for the proteins that participate in fusions events can be used in molecular modelling studies to further explore the potential protein-protein interactions, and to design specific inhibitors which block such interactions, as potential drugs to combat trypanosomiasis. (PDF) [file pone.0068854.s007.pdf]

Fusion Event	Proteins	Domains that hit	Domain ID %	Residue range	Fusion domain
13094041	XP_001218898.1	2ctw	29	124 to 214	125 to 233
	XP_829571.1	2q2g	34	142 to 316	166 to 311
21960686	XP_001218898.1	2ctw	29	124 to 214	125 to 266
	XP_823081.1	2q2g	32	164 to 333	194 to 335
49182202	XP_846892.1	2j89	36	4 to 173	6 to 166
	XP_829255.1	3cez	38	60 to 182	61 to 179
49181135	XP_845692.1	2ctw	33	362 to 448	372 to 542
	XP_823081.1	2q2g	32	164 to 333	195 to 331
17983784	XP_822465.1	1f60	75	2 to 431	3 to 432
	XP_828437.1	1yr6	25	6 to 248	6 to 111
7189996	XP_847169.1	1yq3	42	22 to 140	29 to 138
	XP_826981.1	1kf6	27	72 to 174	69 to 174
148551659	XP_846892.1	2j89	36	4 to 173	9 to 171
	XP_829255.1	3cez	38	60 to 182	67 to 179
297255006	XP_822277.1	2aus	40	43 to 360	277 to 346
	XP_844534.1	1ixk	40	148 to 455	226 to 454
23497583	XP_828707.1	2j0e	99	2 to 264	40 to 171
	XP_822502.1	2bh9	49	71-543	65 to 533
TGME49_091930	XP_827326.1	no templates	-	-	17 to 317
	XP_845376.1	2ghp	12	157 to 448	284 to 447
TGME49_034510	XP_846297.1	1hbk	31	4 to 85	6 to 91
	XP_827785.1	3b7b	21	72 to 292	123 to 272
113631616	XP_823399.1	2q2q	53	2 to 260	4 to 201
	XP_823179.1	2c0c	45	1 to 334	4 to 334
113611229	XP_844119.1	no templates	-	-	52 to 123
	XP_829146.1	1JWH	53	24-214	22 to 215
14209584	XP_845265.1	2cr2	22	430 to 552	430 to 549
	XP_827631.1	1nb8	37	138 to 502	134 to 1104
CMM263C	XP_844283.1	2b9s	67	55 to 482	73 to 512
	XP_827055.1	2b9s	69	222 to 273	201 to 273
CMO271C	XP_828209.1	1aq1	37	20 to 324	27 to 222
	XP_828072.1	3coi	41	21 to 360	184 to 320
CMQ255C	XP_845050.1	1a9x	32-34	5 to 763	6 to 1464
	XP_845052.1	1ml4	41	10 to 327	10 to 327
CMT489C	XP_843903.1	1eq3	64	34 to 122	33 to 122
	XP_845469.1	2qgq	27	208 to 483	55 to 483
15718120	XP_845777.1	3btx	14	230 to 434	228 to 452
	XP_822990.1	3bxo	16	36 to 266	29 to 261
14625283	XP_829015.1	2cqf	25	164 to 202	123 to 207
	XP_828952.1	2db3	36	106-501	99 to 498
14573988	XP_844668.1	no templates	-	-	50 to 417
	XP_846985.1	2i4i	43	62 to 457	59 to 448
7332076	XP_846183.1	1ozn	21	56 to 256	57 to 254
	XP_828320.1	2cxi	30	2 to 387	116 to 298
3881810	XP_846301.1	2bec	19	762 to 915	765 to 832
	XP_823096.1	1b7t	20	17 to 162	79 to 160
2315645	XP_827599.1	1VL8	34	72-216	64 to 141
	XP_951693.1	1ipe	25	2 to 241	94 to 240
2291243	XP_823206.1	2o37	42	1 to 70	6 to 209
	XP_823081.1	2q2g	32	164 to 333	197 to 320
1280169	XP_843940.1	2k3k	38	3 to 88	4 to 87
	XP_845045.1	2cpz	31	419 to 503	424 to 500
Q5XJ54	XP_844769.1	1a8l	13	20 to 205	5 to 220
	XP_803662.1	1wik	33	107 to 209	115 to 204
Q08C92	XP_844345.1	1amo	26	7 to 609	7 to 134
	XP_803801.1	2yx0	32	8 to 330	2 to 377
Q7ZW29	XP_828351.1	no templates	-	-	372 to 503
	XP_846079.1	1fy7	36	130 to 443	134 to 442

Q8JHH7	XP_829376.1	2UZ8	26	185-334	132 to 332
	XP_845565.1	1GAX	39	11-971	11 to 129
B0S700	XP_822515.1	2cg9	26	14 to 132	6 to 122
	XP_823281.1	no templates	-	-	35 to 229
Q1ED17	XP_844283.1	2b9s	67	55 to 482	58 to 499
	XP_827055.1	2b9s	69	222 to 273	193 to 273
Q7T3F6	XP_829326.1	1zc3	23	403 to 504	403 to 512
	XP_827443.1	1z2q	43	9 to 80	11 to 132
Q6DBR7	XP_001219015.1	2yrt	44	107 to 176	109 to 303
	XP_001219018.1	1wh0	20	15 to 113	16 to 107
A8E528	XP_822480.1	3L0W b	27	29-91	16 to 103
	XP_826990.1	1t9z	22	39 to 263	39 to 199
57226667	XP_829573.1	1IUQ	30	77-137	2 to 437
	XP_951511.1	3c48	18	3 to 187	79 to 255
57223091	XP_845050.1	1a9x	32-34	5 to 763	7 to 1430
	XP_845052.1	1ml4	41	10 to 327	1 to 327
RO3T_01042	XP_846297.1	1hbk	31	4 to 85	6 to 80
	XP_828884.1	1qym	26	27 to 139	20 to 106
RO3T_02488	XP_829472.1	3c5v	30	44 to 313	139 to 311
	XP_843679.1	1b43	37	19 to 349	8 to 371
RO3T_04092	XP_847085.1	1pg3	49	26 to 669	155 to 666
	XP_823221.1	1b78	37	105 to 285	101 to 287
RO3T_05874	XP_827933.1	2o95	45	47 to 231	40 to 326
	XP_822660.1	2zkr	62	11 to 111	11 to 114
RO3T_06091	XP_845050.1	1a9x	32-34	5 to 763	7 to 1443
	XP_845052.1	1ml4	41	10 to 327	13 to 327
RO3T_09902	XP_951738.1	1sxj/1um8	13/17	174 to 400/ 561 to 826	170 to 856
	XP_844286.1	1CIQ	29	11-285	36 to 287
RO3T_11245	XP_823179.1	2c0c	45	1 to 334	3 to 307
	XP_845051.1	2aee	24	261 to 454	268 to 405
RO3T_16834	XP_001219015.1	2yrt	44	107 to 176/ 240 to 304	112 to 305
	XP_001219018.1	1wh0	20	15 to 113	15 to 110
Afua_1g10830	XP_843817.1	1euc	67	7 to 301	5 to 301
	XP_822976.1	1euc	47	109 to 505	223 to 496
Afua_1g11540	XP_001219015.1	2yrt	44	107 to 176/ 240 to 304	112 to 298
	XP_001219018.1	1wh0	20	15 to 113	15 to 105
Afua_3g03970	XP_828738.1	1awp	38	4 to 88	6 to 92
	XP_828456.1	1i7p	42	33 to 306	36 to 306
Afua_6g11310	XP_845050.1	1a9x	32-34	5 to 763	4 to 1430
	XP_845052.1	1ml4	41	10 to 327	8 to 311
